# The Impact of Intolerance of Uncertainty on Test Anxiety: Student Athletes During the COVID-19 Pandemic

**DOI:** 10.3389/fpsyg.2021.658106

**Published:** 2021-06-03

**Authors:** Guilin Li, Jie Zhou, Gang Yang, Bing Li, Qing Deng, Liya Guo

**Affiliations:** ^1^College of Physical Education, Sports Psychology and Education Research Center, Southwest University, Chongqing, China; ^2^School of Smart Healthcare Industry, Chongqing City Management College, Chongqing, China; ^3^College of General Education, Chongqing Business Vocational College, Chongqing, China

**Keywords:** COVID-19 pandemic, student athletes, intolerance of uncertainty, test anxiety, mediating effect

## Abstract

Test anxiety caused by intolerance of uncertainty has a negative impact on the physical and mental health of student athletes, especially in the context of the coronavirus disease 2019 (COVID-19) pandemic. A total of 556 grade three high school student athletes in Chongqing, China, were investigated using the Test Anxiety Scale (TAS), Intolerance of Uncertainty Scale-12 (IUS-12), Perceived Social Support Scale, and Coping Style Scale for Middle School Students. Results reveal that more than half the student athletes experienced test anxiety, and the severity was above average during the COVID-19 pandemic. There was a significant correlation between intolerance of uncertainty, perceived social support, coping style, and test anxiety. A positive correlation was found between test anxiety, intolerance of uncertainty, and coping style toward emotions, and a negative correlation between test anxiety, perceived social support, and coping style toward problems. Intolerance of uncertainty has a direct predictive effect on test anxiety, and perceived social support and coping style play a chain mediator role between intolerance of uncertainty and test anxiety. By constructing the mediating effect model, we can, to some extent, reveal the mechanism of the influence of intolerance of uncertainty on test anxiety. This study has a certain reference value for the prevention of test anxiety in student athletes in the context of the COVID-19 pandemic.

## Introduction

At the end of 2019, the coronavirus disease 2019 (COVID-19) was first identified, and then spread globally in a short span of time. The World Health Organization (WHO) announced that COVID-19 has become a public health emergency of international concern and declared it a global pandemic. This newly emerging, highly pathogenic infectious disease is highly contagious, with a wide range of transmission routes, high fatality rates, and a high degree of uncertainty of risk sources (Sun and Zhou, 2020). Affected by the COVID-19 pandemic, countries around the world executed preventative and control measures such as home quarantines, the shutdown of non-essential services, school suspensions and keep social distancing guidelines, to curb the spread of the virus. In China, for example, the Ministry of Education issued a notice that the National College Entrance Examination will be postponed for one month in 2020, and the dates of college entrance examination for sports major students^1^ are yet to be determined. College application is a life-changing opportunity for many student athletes. When the epidemic will end, whether the college entrance examination for sports major students can be held as scheduled, and how to guarantee the physical and mental health of students athletes under the normal prevention and control during the epidemic period have become the hot topics of social concern.

Every aspect of human daily life is characterized by uncertainty, especially in the event of a major natural disaster or sudden public health event (Rosen et al., 2014). Uncertainty refers to a psychological state caused by an individual's inability to accurately know an event or a decision result (Carleton et al., 2012). For individuals, the future is mostly uncertain. However, if the uncertainty of this scenario is prolonged and significant, it will undoubtedly have a serious impact on an individual's psychology. Intolerance of uncertainty (IU) is conceptualized as a cognitive bias that affects how a person perceives, interprets, and responds to uncertain situations on a cognitive, emotional, and behavioral level (Freeston et al., 1994; Dugas et al., 2004). Individuals high in IU react negatively to uncertainty, believe they are unable to cope with ambiguous situations, and consider uncertainty itself threatening (Carleton et al., 2007; Luhmann et al., 2011; Jensen et al., 2014). Today, IU has become a popular concept to explain anxiety, many studies have found robust associations between IU and various anxiety-related conditions, and differences in an individual's ability to tolerate uncertainty affects the level of anxiety (Nicholas et al., 2007; Boelen and Reijntjes, 2009; Carleton et al., 2013; Norr et al., 2013; Oglesby and Schmidt, 2017). Anxiety is a typical negative emotional state of people facing emergencies, such as fear of infection in the case of major infectious diseases, e.g., COVID-19 (Cao et al., 2020), EBoV (Bah et al., 2020), H1N1 (Taha et al., 2014), and SARS (Li et al., 2004).

As a special form of anxiety, test anxiety refers to physical and mental changes, such as nervousness, anxiety, restlessness, and insomnia, that occur when individuals face taking an exam. Here, they desire good grades and fear failure (Sarason, 1980; Whitaker Sena et al., 2007). It has become one of the most prominent psychological disorders among middle school students (Aysan et al., 2001). As a special group in high school, student athletes shoulder the responsibility of both cultural learning and professional training simultaneously. They have a strong sense of purpose upon entering college and have the dual task of learning and training. For most student athletes, it is inevitable that they will experience a certain degree of anxiety in the face of the college entrance examination, which would be the most important exam in their life. However, excessive anxiety will not only have a negative impact on the training, learning efficiency, and test performance but also cause serious harm to their physical and mental development. The significant correlation between IU and test anxiety has been investigated in the literature. Ryzewicz (2008) found that IU could significantly predict test anxiety and suggested that students' intolerance to the uncertainty of exam results was a possible reason. In Christopher et al.'s (2020) research, they pointed out that IU was significantly and positively associated with both trait and state test anxiety, which suggests IU plays an important contributory role in test anxiety. However, the impact of COVID-19 on the relationship between IU and test anxiety is still unknown.

In addition to directly affecting test anxiety, IU may also indirectly cause anxiety through mediating variables. Mishel (1988) proposed the theory of disease uncertainty, believing that social support is an important part of providing help. Social support refers to an individual's experience or satisfaction of being valued, respected, supported, and understood by others (Barrera, 1986; MdYasin and Dzulkifli, 2010). It may come from different sources such as family, friends, teachers, community, or any social groups to which one is affiliated. Social support can come in the form of tangible assistance provided by others or in the form of perceived social support that assesses individuals' confidence of the availability of adequate support when needed (Hengl, 1997). Previous research shows that low social support is one of the predictors of psychological problems and associated with depression and anxiety (Teoh and Rose, 2001), it is considered as a mechanism to buffer against life stress (Steese et al., 2006). Wang et al.'s (2014) empirical research showed that perceived social support can predict students' test anxiety, and the lower the level of perceived social support, the more likely the student is to experience test anxiety.

Coping style refers to the cognitive strategies and behavioral styles adopted by individuals in the face of an emergency stimulus or stress environment to reduce the negative effects of stress, and is an important mediating factor in the process of psychological stress (Skinner et al., 2003). Spielberger and Vagg (1995) point out that after individuals appraise a testing situation as stressful, they employ coping styles to manage their level of test anxiety. According to the impact of coping styles on the body and mind, they can be divided into two types: problem-oriented coping and emotion-oriented coping (Lazarus and Folkman, 1984). A problem-oriented coping style involves engaging in behaviors to overcome the problem causing distress (e.g., seeking social support, devising a plan to study for a stressful exam and cognitive reconstruction) whereas emotion-oriented coping is an attempt to regulate emotions that are evoked by the stressful event through fantasy, avoidance, denial, and other strategies. Daisy and Richard (2019) found that students experience less test anxiety as they use less avoidant emotion-oriented coping. Arana and Furlan (2016) pointed out that problem-focused coping explained the relationships between adaptive/maladaptive perfectionism and test anxiety.

According to stress theory, the intensity of the stressor, perceived social support, and coping style are important factors in evaluating whether stress can damage one's health (Lazarus and Folkman, 1984; Schwarzer and Knoll, 2007). After an individual encounters a stressful event, perceived social support and coping style become an important moderators or mediators between stress and consequences. Facing major stress events, perceived social support and coping style—as important influencing factors of psychological stress—play crucial roles in individual mental health. Roohafza et al. (2014) shows that active coping styles and perceived social supports are protective factors for depression and anxiety. Perceived social support can directly promote individuals' positive response, and it can also indirectly promote individuals' positive responses by buffering fear, anxiety, and other psychological stressors (Degroote et al., 2014). To the best of our knowledge, the internal mechanism among IU, test anxiety, perceived social support, and coping style is still an open problem.

**Figure 1:**
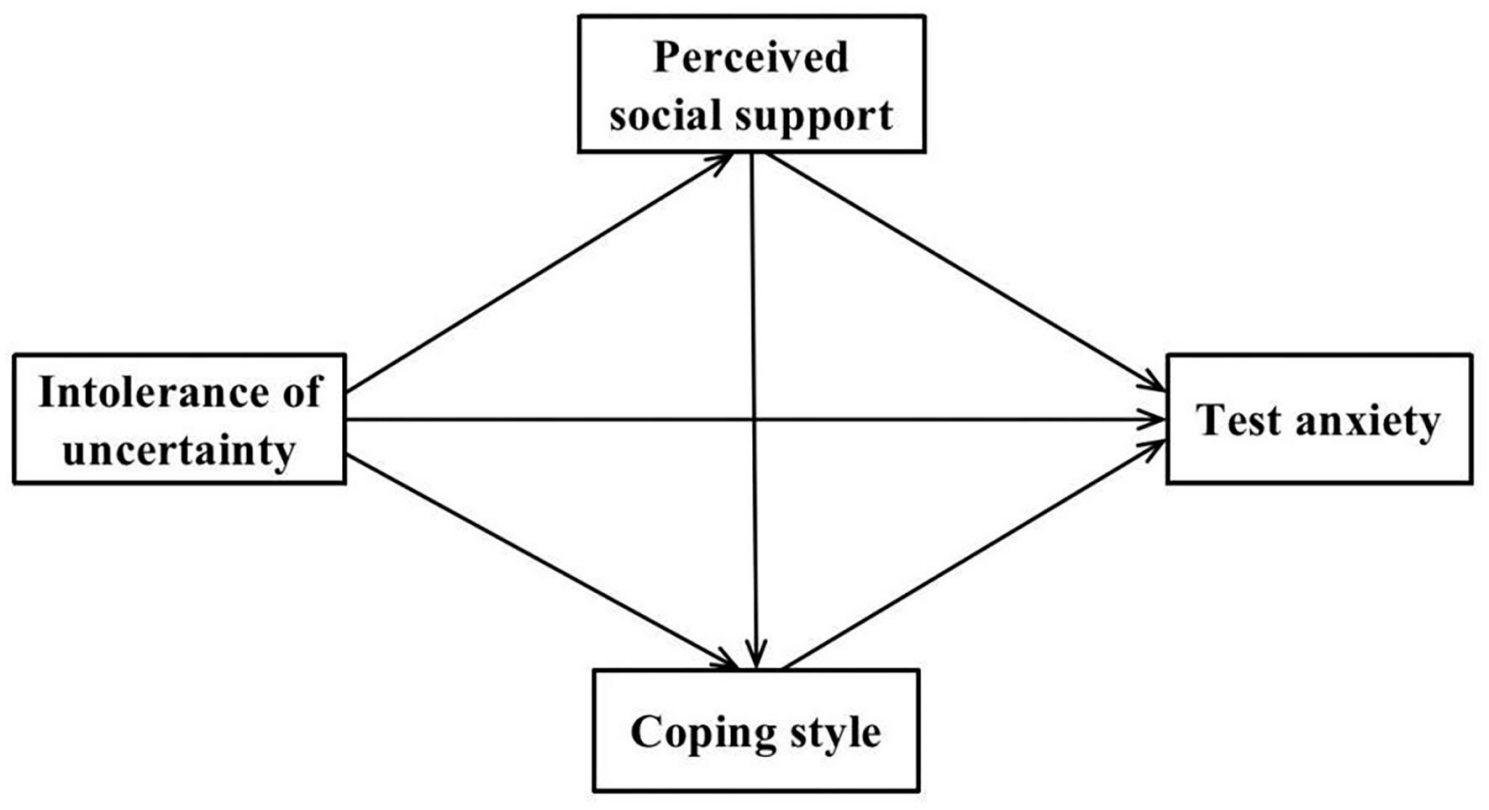
The hypothesized mediation model.

COVID-19 is highly unpredictable, and its risk sources are mostly from uncertain natural factors. With the continuation of the pandemic, student athletes will face a long period of closed study, training, and social isolation. It can be reasonably speculated that test anxiety of student athletes will continue to increase under the dual pressure of the college entrance examination/cultural examination and professional skills examination. In this paper, the authors discuss the impact of IU on test anxiety of student athletes, by considering the current pandemic situation and the great degree of uncertainty of sports major enrollment examinations as the entry point. Four hypotheses have been proposed: H1: IU can predict test anxiety directly and positively; H2: Perceived social support plays a mediating role in the relationship between IU and test anxiety; H3: Problem-oriented coping style mediates the relationship between IU and test anxiety positively, while emotion-oriented coping style mediates it negatively; H4: Perceived social support and coping styles play a chain mediating role between IU and test anxiety, as shown in Figure 1.

By verifying the above hypotheses, this study reveals the internal mechanism of the test anxiety of student athletes and provide certain decision-making reference points and theoretical support for the prevention of and interventions for test anxiety during the pandemic.

## Materials and Methods

### Participants

This study adopted random cluster sampling and selected student athletes from 25 middle schools in Chongqing, China, as participants to complete a survey questionnaire, the participants was limited to junior high school athletes who will take the sports major enrollment examinations this year. Initially, A total of 587 questionnaires were distributed, however, 18 participants did not completed the study, and 13 were deemed invalid (such as all the items checked in the questionnaire were the same, the question is not a multiple choice, but two or more options have been checked, there is a contradiction between the positive and negative questions), resulting in the final sample size of 556, with an effective recovery rate of 94.7%. Among the participants, *n* = 313 were male (56.3%) and *n* = 243 were female (43.7%); *n* = 403 were recent graduates students (72.5%) and *n* = 153 were former graduates students (27.5%); *n* = 317 participants registered for the National Sports Major Entrance Test (57.0%), *n* = 79 participants for the College Entrance Examination of High Level Athletes (14.2%), *n* = 160 participants for the National Physical Education Single Entrance Examinations (28.8%); According to Chinese technical classification of athletes, *n* = 247 participants were classified as Level 3 and below athletes (9.7%), *n* = 255 as Level 2 athletes (45.9%), *n* = 54 as Level 1 or Master Level (9.7%), the higher the level in turn (Gazette of the State Council of the People's Republic of China, 1995). Their average age was *M* = 17.63 years (SD = 3.147). Written and informed consent was obtained from the parents/legal guardians of all non-adult participants.

### Procedure

Permission was obtained from the Ethics Committee of the Department of Psychology, Southwest University. All the data were collected offline from 20 April 2020 to 26 April 2020, during the first week after resuming school following the quarantine caused by the COVID-19 pandemic. Due to the COVID-19 epidemic, all the schools under investigation are fully closed management and cannot distribute questionnaires to each student athlete in person. The researchers contacted with the school managers and coaches, after the permission of them, mailed the questionnaires to the coaches, invited them to help distribute and collect questionnaires, and supervised the student athletes to complete the questionnaires carefully. Prior to answering the items, participants read information about the purpose of the study, implications of participation, and data protection. The information stressed that participation was completely voluntary and anonymous. The questionnaire took about 20 min to complete.

### Measures

#### Test Anxiety Scale

This study adopted the Chinese version of the Test Anxiety Scale (TAS), translated and revised by Wang (2001) based on the original compiled by Sarason (1998), an American clinical psychologist. The scale explores individual attitudes toward tests, various psychological states, and physical discomfort before and after a test. It includes 37 questions, each of which requires a yes or no answer; “yes” is scored as 1 point and “no” is scored as 0 points. Questions 3, 15, 27, 29, and 33 are reverse scored. The total score is calculated to evaluate test anxiety levels. The score range is 0–37, and the higher the test score, the higher the anxiety level. A total score of 15 or above indicates that a respondent clearly feels a certain degree of discomfort caused by taking exams, which can determine the existence of test anxiety (Newman, 1996). The internal consistency of the scale was good (Cronbach's α = 0.83).

#### Intolerant of Uncertainty Scale (IUS-12)

This study adopted the short version of the Intolerance of Uncertainty Scale (IUS-12), originally compiled by Freeston et al. (1994) and revised by Wu et al. (2016). This scale measures the degree of aversion of individuals to perceived uncertain information. There are 12 items in total, and a 5-point Likert scale is used for scoring (1 = highly inconsistent to 5 = highly consistent). The scale is divided into two subdimensions, of which seven items are “predictive anxiety,” referring to worrying and fear of future events while five other items are “inhibitive anxiety,” referring to the avoidance and suppression of uncertainty. Higher scores indicate higher tolerance for uncertainty. In this study, Cronbach's α coefficient was 0.93 for the total questionnaire and 0.91 and 0.88 for the subdimensions. The confirmatory factor analysis results were as follows: X^2^/df = 2.82, RMSEA = 0.06, AGFI = 0.94, CFI = 0.97, TLI = 0.97, IFI = 0.97, and GFI = 0.96, indicating that the questionnaire had good model fit.

#### Coping Style Scale for Middle School Students

In this study, the Coping Style Scale for Middle School Students, compiled by Zheng and Chen (2000), was used. The scale has two subscales and consists of 36 items rated on four levels score. One is the subscale of “problem-oriented coping,” including “problem solving,” “seeking social support,” and “positive rationalization,” with a total of 19 items. The other is the subscale of “coping toward emotion,” including “patience,” “escape,” “vent emotion,” and “fantasy denial,” with a total of 17 items. After the internal consistency test, 11 items were eliminated because of their small contribution. For the problem-oriented coping subscale, Cronbach's α coefficient was 0.94 after deleting items 3, 7, 13, 22, 28, and 34. Cronbach's α coefficient was 0.93 after deleting items 14, 21, 26, 30, and 35 on the emotions-oriented coping subscale. The score is added to the factor score belonging to the same subscale, and the subscale score is generally excluded from the total score of the scale. The corresponding measurement model verifies the results for problem-oriented coping, X^2^/df = 2.82, RMSEA = 0.06, AGFI = 0.93, CFI = 0.95, TLI = 0.97, IFI = 0.97, and GFI = 0.95, and emotion-oriented coping, X^2^/df = 2.47, RMSEA = 0.05, AGFI = 0.94, CFI = 0.98, TLI = 0.97, IFI = 0.98, and GFI = 0.96, indicating that the questionnaire had good model fit.

#### Perceived Social Support Scale

This study adopted the Perceived Social Support Scale, originally compiled by Zimet and Dahlem (1988) and revised by Jiang (2001). This scale has 12 questions across three subscales: family support, friend support, and other support. Each subscale has four questions. The average score of each item reflects the total degree of social support felt by the respondent. The higher the score, the higher the total degree of social support the respondent feels. Responses are scored on a 7-point Likert scale, ranging from “1 = strongly disagree” to “7 = strongly agree.” In this study, Cronbach's α coefficient was 0.90 for the total questionnaire and 0.90, 0.87, and 0.81 for the subdimensions of family support, friend support, and other support, respectively. The confirmatory factor analysis results were as follows: X^2^/df = 2.17, RMSEA = 0.05, AGFI = 0.95, CFI = 0.98, TLI = 0.98, IFI = 0.98, and GFI = 0.97, indicating that the questionnaire had good model fit.

### Data Analysis

In this study, SPSS 21.0 was used for statistical analysis (including reliability and validity test, descriptive statistics, an independent sample *t*-test, one-way ANOVA, correlation analysis, and variance analysis). AMOS21.0 was adopted when establishing the structural model, based mainly on the mediation effect test by Wen et al. (2004).

## Results

### Analysis of Test Anxiety During the COVID-19 Pandemic

This study used descriptive statistics, independent sample *t*-tests, and one-way ANOVA to analyze the differences in demographic factors of student athletes' test anxiety in order to understand their level of test anxiety during the COVID-19 pandemic. As shown in Tables 1, 2, the mean test anxiety score of the 556 student athletes was 16.11 ± 7.22, and those with a TAS score ≥ 15 were considered to have test anxiety. It was found that 314 of the student athletes had test anxiety, accounting for 56.5% of the total participants.

**Table 1:** Analysis on the differences in test anxiety among student athletes with different demographic characteristics.

**Project**	**Grouping**	**N**	**M**	**SD**	**T/F**
Gender	Male	313	15.56	7.47	−2.04^*^
	Female	243	16.81	6.82	
Examinee category	Fresh graduates	403	15.84	7.25	−1.44
	Previous life	153	16.82	7.11	
Exam type	National Sports Major Entrance Test	317	17.00	7.20	5.62^**^
	College Entrance Examination of High Level Athletes	79	14.59	6.54	
	National Physical Education Single Entrance Examinations	160	15.13	7.35	
Athlete level	Master and Level 1	54	15.24	6.89	10.94^***^
	Level 2	255	14.77	7.21	
	Level 3 and below	247	17.68	7.00	

* *P < 0.05*,

** *P < 0.01*,

*** *P < 0.001, the same as below*.

**Table 2:** Mean, standard deviation, and Pearson correlation coefficient between the variables in each study (*n* = 556).

	**M**	**SD**	**1**	**2**	**3**	**4**	**5**	**6**	**7**	**8**
1. IU	30.67	10.89	1							
2. Perceived social support	54.69	12.96	−0.32***	1						
3. Family support	18.98	5.61	−0.30***	0.83***	1					
4. Friends support	17.77	5.46	−0.26***	0.84***	0.56***	1				
5. Other support	17.95	4.99	−0.22***	0.75***	0.41***	0.45***	1			
6.Problem-oriented coping	33.87	10.11	−0.41***	0.36***	0.27***	0.31***	0.29***	1		
7.Emotion-oriented coping	25.11	8.40	0.37***	−0.38***	−0.31***	−0.30***	−0.30***	−0.31***	1	
8.Test anxiety	16.11	7.22	0.47***	−0.41***	−0.35***	−0.34***	−0.29***	−0.43***	0.42***	1

To further explore the current situation of student athletes' test anxiety, this study examined the differences in demographic variables such as gender, examinee category, exam type, and athlete level. The results showed that there were gender differences in test anxiety [*T*_(554)_ = −2.04, *p* < 0.05], as the test anxiety level of female students was higher than that of male students. There were significant differences in test anxiety among the different types of examinees [*F*_(2)_ = 5.62, *p* < 0.01], among which the anxiety level was the highest (17.00 ± 7.20) for students taking the National Sports Major Entrance Test, followed by those taking the National Physical Education Single Entrance Examinations (15.13 ± 7.35). The anxiety level was the lowest (14.59 ± 6.54) for students taking the High-Level Entrance Examination. The test anxiety of student athletes at different athletic levels was significantly different [*F*_(2)_ = 10.94, *p* < 0.001]. The test anxiety level of the student athletes with Level 3 and below was the highest (17.68 ± 7.00), followed by athletes at Master and Level 1 (15.24 ± 6.89), Level 2 athletes ranked the lowest (14.77 ± 7.21).

### Descriptive Statistics and Correlation Analysis of Study Variables

The correlation analysis of IU, perceived social support, coping style and test anxiety of student athletes showed that there were significant correlations among all variables (Table 2). Test anxiety was significantly positively correlated with IU and emotion-oriented coping (*r* = 0.42 ~ 0.47, *p* < 0.001), and significantly negatively correlated with perceived social support and its three dimensions as well as problem-oriented coping (*r* = −0.29 ~ −0.43, *p* < 0.01). This indicated that all influencing factors of test anxiety. Thus, the higher student athletes' level of test anxiety, the higher their IU level, the less social support they felt, and the more likely they were to adopt an emotion-oriented negative coping style. IU was significantly negatively correlated with perceived social support and problem-oriented coping (*r* = −0.32 ~ −0.41, *p* < 0.001), and significantly positively correlated with emotion-oriented coping (*r* = 0.37, *p* < 0.001). Perceived social support was significantly positively correlated with problem-oriented coping (*r* = 0.36, *p* < 0.001) and negatively correlated with emotion-oriented coping (*r* = −0.38, *p* < 0.001). There was a significant negative correlation between problem-oriented coping and emotion-oriented coping (*r* = −0.31, *p* < 0.001). The correlation coefficient among all the study variables was significant, which provided a certain prerequisite for the subsequent mediating effect test.

### Mediating Effect Test

First, we conducted regression analysis on each study variable, the results are shown in Table 3. In Item 1, when IU was used as a predictor variable, and perceived social support, problem-oriented coping, and emotion-oriented coping were the dependent variables, IU for perceived social support (β = −0.32, *p* < 0.001) and problem-oriented coping (β = −0.41, *p* < 0.001) significantly negatively predicted test anxiety, while emotion-oriented coping (β = 0.37, *p* < 0.001), significantly positively predicted test anxiety. In Item 2, when IU was the predictor variable and test anxiety was the dependent variable, IU had a significant positive predictive effect on test anxiety (β = 0.47, *p* < 0.001); thus, H1 was supported. In Item 3, test anxiety was used as the dependent variable when problem-oriented coping, emotion-oriented coping, perceived social support, and IU were used as predictor variables simultaneously, all predictor variables could significantly predict test anxiety. Problem-oriented coping (β = −0.20, *p* < 0.001) and perceived social support (β = −0.18, *p* < 0.001) negatively predicted test anxiety, while emotion-oriented coping (β = 0.19, *p* < 0.001) and IU (β = 0.26, *p* < 0.001) positively predicted test anxiety. However, IU's predictive power for test anxiety decreased, suggesting that perceived social support and coping style played a partial mediating role in the relationship between IU and test anxiety.

**Table 3:** Mediating regression analysis.

	**The dependent variable**	**The independent variable**	**B**	**SE**	**β**	**t**	***R*^**2**^**	**F**
Item 1	Perceived social support	IU	−0.39	0.05	−0.32***	−8.06	0.11	64.92***
	Problem-oriented coping	IU	−0.38	0.04	−0.41***	−10.46	0.17	109.40***
	Emotion-oriented coping	IU	0.29	0.03	0.37***	9.42	0.14	88.79***
Item 2	Test anxiety	IU	0.31	0.03	0.47***	12.54	0.22	157.16***
Item 3	Test anxiety	Problem-oriented coping	−0.14	0.03	−0.20***	−5.18	0.36	78.34***
		Emotion-oriented coping	0.17	0.03	0.19***	5.00		
		Perceived social support	−0.10	0.02	−0.18***	−4.63		
		IU	0.17	0.03	0.26**	6.61		

To further verify the mediating effects of perceived social support and coping style on the relationship between IU and test anxiety, a structural equation modeling was used to analyze the relationships between variables. In the analysis, IU was used as the independent variable in the model, test anxiety as the dependent variable, and perceived social support and coping style as the mediating variable. According to the mediating effect test steps, the direct effect of IU on test anxiety was tested first, and then the two mediating variables of perceived social support and coping style were added to determine the significance of the model fit and path coefficient (Fang, 2012). The fit indices of the structural equation model were X^2^/df = 1.87, RMSEA = 0.04, AGFI = 0.97, CFI = 0.99, TLI = 0.98, IFI = 0.99, and GFI = 0.99, indicating the model has a good degree of fit and is suitable for test of the mediating effects.

**Figure 2:**
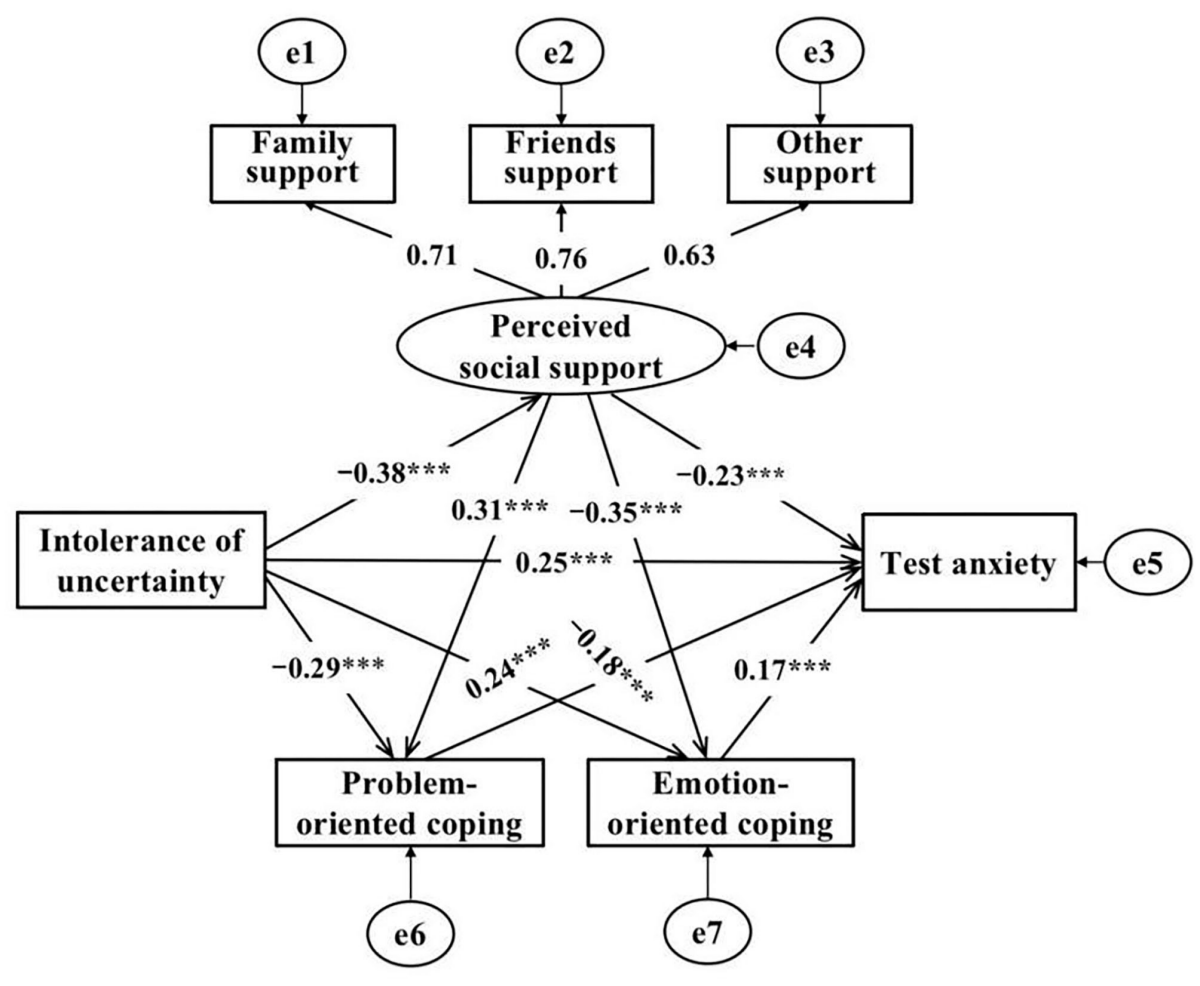
The mediating model of perceived social support, coping styles between IU and test anxiety.

The structural equation model (Figure 2) showed that IU positively predicted test anxiety (β = 0.25, *p* < 0.001) and emotion-oriented coping (β = 0.24, *p* < 0.001), and negatively predicted perceived social support (β = −0.38, *p* < 0.001) and problem-oriented coping (β = −0.29, *p* < 0.001). Perceived social support negatively predicted test anxiety (β = −0.23, *p* < 0.001) and emotion-oriented coping (β = −0.35, *p* < 0.001), and positively predicted problem-oriented coping (β = 0.31, *p* < 0.001). After adding the mediating variable, the path coefficients still reached the level of significance, indicating the existence of a mediating effect. The specific results were as follows: (1) the mediating effect of “IU → perceived social support → test anxiety” was significant, the bootstrap confidence interval did not contain 0, and the standardized effect value was 0.09, accounting for 19.15% of the total effect;(2) the mediating effect of “IU → problem-oriented coping → test anxiety” was significant, the bootstrap confidence interval did not contain 0, and the standardized effect value was 0.05, accounting for 10.64% of the total effect; (3) “IU → emotion-oriented coping → test anxiety” had a significant mediating effect, with an effective value of 0.04, accounting for 8.51% of the total effect, the bootstrap confidence interval did not contain 0; (4) “IU → perceived social support → problem-oriented coping → test anxiety” had a significant chain mediating effect, with an effective value of 0.02, accounting for 4.26% of the total effect, the bootstrap confidence interval did not contain 0; and (5) “IU → perceived social support → emotion-oriented coping → test anxiety” had a significant chain mediating effect, with an effective value of 0.02, accounting for 4.26% of the total effect, the bootstrap confidence interval did not contain 0 (Table 4). Therefore, H2, H3, and H4 were all supported.

**Table 4:** Mediation effect analysis.

**Path of influence**	**Standardized effect value**	**The ratio of the total effect**	**Bootstrap 95% confidence interval**	**Significant**
The total effect	0.47	—	(0.43,0.49)	Significant
Direct effect	0.25	53.19%	(0.21,0.28)	Significant
Total mediating effect	0.22	46.81%	(0.20,0.24)	Significant
Path 1	0.09	19.15%	(0.08,0.11)	Significant
Path 2	0.05	10.64%	(0.03,0.06)	Significant
Path 3	0.04	8.51%	(0.02,0.05)	Significant
Path 4	0.02	4.26%	(0.01,0.04)	Significant
Path 15	0.02	4.26%	(0.01,0.03)	Significant

*Path 1: IU→ perceived social support→ test anxiety; Path 2: IU→ problem-oriented coping→ test anxiety; Path 3: IU→ emotion-oriented coping→ test anxiety; Path 4: IU→ perceived social support→ problem-oriented coping→ test anxiety; Path 5: IU→ perceived social support→ emotion-oriented coping→ test anxiety*.

## Discussion

### Current Situation of Test Anxiety Levels

The results of this study show that the incidence of test anxiety (≥15 points) among student athletes is 56.5%. Thus, more than half of these students experience test anxiety, which cannot be ignored. In addition, this study found that there were significant gender differences in test anxiety, with female students having a higher anxiety level than male students. Most girls are sensitive and prone to emotional fluctuations, and are also more likely to have negative emotions, such as tension and anxiety, toward exam stress. There were also significant differences in test anxiety among students with different exam types and athletic levels. This result may be due to the fact that students who take the National Physical Education Single Entrance Examination and College Entrance Examination of High Level Athletes are all at least at Level 2 and above. Their professional motor skill level is relatively high, and they also have certain experience with competition. However, the students who take the National Sports Major Entrance Test have relatively low motor skills, and lack competition experience. Furthermore, students must pass both cultural and athletic tests to admit to universities, and the cultural admission score for the former is lower. Therefore, most of the latter ones are under great pressure before the exam, and their psychological process is relatively complex; thus, their anxiety level is relatively high.

### Direct Impact of IU on Test Anxiety

This study found that IU had a direct predictive effect on test anxiety; that is, individuals with high IU showed high test anxiety. Candidates with high IU generally regard an exam situation as uncertain (Ryzewicz, 2008). They feel uncertain about what will happen during the exam and their performance results, which leads to worry and anxiety. Examinations are critical events in students' lives (Ladouceur et al., 2000). High IU individuals tend to experience uncertainty and stress events for prospective disaster interpretation. They expect adversity and mentally exaggerate the anticipated possibility for and severity of disaster. They think they cannot cope with the situation, and this produces higher levels of test anxiety (Dugas et al., 2005). In the context of the COVID-19 pandemic, student athletes have shown higher levels of test anxiety in the face of uncertain future scenarios.

### Mediating Effects of Perceived Social Support and Coping Style

This study found that perceived social support played a mediating role between IU and test anxiety among student athletes. First, perceived social support can negatively predict test anxiety. Second, perceived social support plays the role of a “bridge” between IU and the test anxiety. In other words, perceived social support mediates the relationship between IU and test anxiety. In uncertain circumstances, if student athletes obtain more spiritual support and encouragement from their family, teachers, and coaches, as well as classmates, friends, and other support groups, their psychological regulation ability will become stronger and they will be less likely to experience test anxiety.

The results of this study also showed that positive problem-oriented coping styles could negatively predict test anxiety, while negative emotion-oriented coping styles could positively predict test anxiety. Coping styles played a mediating role between IU and test anxiety. Faced with the same examination pressure, different coping styles affect individual psychological states in the stress process, thus affecting the individual stress responses, which may eventually lead to different response results. In an uncertain situation, student athletes with test anxiety were more likely to deal with exam stress using patience, escape, venting emotions, and fantasy denial, and less likely to deal with stress by means of problem-solving, thus making the situation worse. The more affected by the negative emotions, the stronger the test anxiety.

This study further found the chain mediating effect of perceived social support and coping style between IU and test anxiety. Specifically, under the stimulation of uncertain stress events, IU indirectly affected the coping style (problem-oriented/emotion-oriented) through perceived social support, and then acted on the level of individual test anxiety. It confirmed the main effect model of perceived social support (Lutz and Lakey, 2001) and the buffer model (Cohen and Wills, 1985). Improved perceived social support can effectively promote students' responses, encourage them to adopt more active coping strategies such as problem-oriented coping, and less negative coping strategies like emotion-oriented coping, which will significantly improve coping effectiveness. This will shield students from negative emotions such as fear and anxiety, thus inhibiting test anxiety.

## Limitations

Although this study explores the influence of IU on test anxiety, and further reveals the internal mechanism of test anxiety, it has the following shortcomings: (1) The cross-sectional study design is adopted in this study, which makes it difficult to make accurate causal inference. Further studies can be conducted through experimental study and follow-up design in the future. (2) The survey is only conducted in one city of China, which cannot be directly generalized to the whole of China. (3) This study only focused on the mediating effect of coping style and perceived social support on the relationship between intolerance of uncertainty and test anxiety, but in reality there are other mediating variables, such as personality traits, self-esteem, self-efficacy, etc., which need to be further studied in the future. Despite these limitations, the results of this study help us understand the intrinsic relationship between IU and test anxiety to a certain extent as well as its possible causes and mechanisms in the context of Chinese culture.

## Conclusions

Taking Chongqing as example, this study examined the test anxiety caused by IU in Chinese student athletes. A mediating effect model between IU and test anxiety had been constructed by introducing the perceived social support and coping style into a structural equation model. This model has good explanatory power for the test anxiety of student athletes, and to a certain extent reveals the mechanism of the uncertainty affecting the test anxiety of student athletes.

Research shows that, schools and education departments should attach greater importance to the test anxiety of student athletes in the context of the COVID-19 pandemic. Psychological counseling and interventions for test anxiety are necessary and urgent. On the basis of strengthening the necessary physical and technical training, attention should be paid to the psychological training of this special examinee group. In the process of prevention and management of test anxiety, factors such as gender and examination category should also be considered. In particular, test anxiety among student athletes could be effectively reduced by following methods: (1) strengthening the level of tolerance for uncertainty; (2) offering more spiritual support and encouragement from their family, teachers, and coaches, as well as classmates, friends, and other support groups; (3) encouraging the adoption of problem-oriented coping styles instead of emotion-oriented coping styles.

## Data Availability Statement

The raw data supporting the conclusions of this article will be made available by the authors, without undue reservation.

## Ethics Statement

The studies involving human participants were reviewed and approved by Ethics Committee of School of Physical Education, Southwest University. Written informed consent to participate in this study was provided by the participants' legal guardian/next of kin.

## Author Contributions

GL, JZ, GY, QD, and LG conceived the study, interpreted the data, drafted and revised the work, approved the final version of the manuscript to be published, and agreed to be accountable for all aspects of the work. All authors contributed to the article and approved the submitted version.

## Conflict of Interest

The authors declare that the research was conducted in the absence of any commercial or financial relationships that could be construed as a potential conflict of interest.

## Footnotes

^1^As an important way to enter University, the college entrance examination for sports major students mainly recruits students with sports expertise. At present, the college entrance examination for sports major students can be roughly divided into three categories: the National Sports Major Entrance Test, College Entrance Examination of High Level Athletes, and National Physical Education Single Entrance Examination.
